# The Genomic Landscape of Cattle: Domestication, Dispersal, and Adaptive Evolution

**DOI:** 10.3390/ani16050776

**Published:** 2026-03-02

**Authors:** Yiduan Liu, Wenbin Dao, Ruixia Gao, Xinyang Fan, Ruifei Yang, Yongwang Miao

**Affiliations:** 1 College of Animal Science and Technology, Yunnan Agricultural University, Kunming 650201, China; liuyiduanabc@163.com (Y.L.); dwbin666@126.com (W.D.); malegreshell@126.com (R.G.); xinyangfan1@ynau.edu.cn (X.F.); 2 Institute of Animal Genetics and Breeding, Yunnan Agricultural University, Kunming 650201, China

**Keywords:** aurochs, domestication, *Bos taurus*, *Bos indicus*, adaptive evolution, ancient DNA, pangenome

## Abstract

Domestic cattle represent one of the most significant evolutionary successes in the history of human–animal mutualism. This review synthesizes evidence from paleogenomics and modern population genetics, particularly recent pangenome analyses, to reconstruct a comprehensive evolutionary trajectory of cattle. We outline the two domestication events: the emergence of taurine cattle (*Bos taurus*) in the Fertile Crescent (~10,500 years ago) and zebu cattle (*Bos indicus*) in the Indus Valley (~8000 years ago). Following domestication, cattle dispersed globally alongside human migration, resulting in a complex genetic mosaic shaped by introgression with wild relatives and extensive admixture between lineages. By integrating data from mitochondrial DNA, Y-chromosome haplotypes, and whole-genome sequencing of modern, ancient, and wild samples, we reconstruct the detailed global dispersal of cattle. Furthermore, we dissect the molecular mechanisms underlying phenotypic diversity, emphasizing how natural selection has driven environmental adaptation, how artificial selection has optimized production traits, and how the emerging bovine pangenome is unveiling “hidden” genetic variations critical for climate resilience and disease resistance. Ultimately, this review summarizes the origin, dispersal, and genomic diversity of cattle, offering vital insights for the conservation of indigenous genetic resources and the advancement of molecular breeding strategies in the face of a changing global climate.

## Simple Summary

From the initial domestication of the aurochs in the Fertile Crescent and Indus Valley to the emergence of specialized breeds such as Simmental and Holstein, cattle have coevolved inextricably with humans. This review highlights that modern cattle are not static entities but dynamic genomic mosaics, shaped by ancient migrations, genetic introgression from extinct wild relatives, and continuous adaptation to human demands and environmental challenges. Understanding this complex history through the lens of modern genomics extends far beyond documenting the diversity of the cattle genome; it provides an essential roadmap for molecular breeding and sustainable livestock systems to respond to the challenge of a changing global climate.

## 1. Introduction

Cattle occupy a unique and pivotal position in both the biological and cultural history of humanity. As a primary source of protein (meat and milk), secondary products (leather, dung for fuel and fertilizer), and draught power, cattle have served as biological engines driving the intensification of agricultural systems for millennia. Beyond their intrinsic utilitarian value, cattle have transcended to become symbols of wealth, social status, and divinity in numerous cultures, deeply integrating into traditional customs and belief systems [1]. Cattle were domesticated via the prey pathway, a gradual transition from the hunting of wild bovids to managed husbandry, thereby securing consistent access to these animal resources [2]. Extensive archaeological and genetic research over the past few decades has elucidated the dual origins of domestic cattle as well as the trajectories of their global dispersal, which closely parallel human migration and population expansion events. To date, over one thousand recognized cattle breeds exist globally, each uniquely shaped by long-term artificial selection and local environmental adaptation [3,4]. However, modern genomics has revealed that contemporary cattle populations are not merely static entities, but rather highly dynamic genomic mosaics. Their genomic diversity has been sculpted by a complex interplay among ancient human-mediated migrations, introgressive hybridization with extinct wild relatives, and continuous adaptive evolution in response to varying selection pressures and environmental extremes. Recent advances in whole-genome sequencing (WGS) technologies, coupled with the development of pangenome graphs that capture the full spectrum of genomic variation beyond single reference genomes, have fundamentally transformed our understanding of bovine genetic diversity and phenotypic variation. This review aims to synthesize the most recent advancements in domestic cattle research, focusing on four core themes: evolutionary origin, global dispersal, environmental adaptation, and artificial selection. Beyond traditional single-nucleotide polymorphism (SNP) analyses, we incorporate emerging findings from bovine pangenome assemblies and structural variation (SV) analyses, which have proven exceptionally powerful in identifying key genetic variants underlying breed-specific adaptations. By providing a comprehensive synthesis of the genetic diversity and population structure of cattle, with a special emphasis on indigenous breeds harboring unique adaptive alleles, this article seeks to establish a robust scientific foundation for the conservation, sustainable utilization, and molecular improvement of local cattle genetic resources. 

## 2. The Dual Origins of Cattle: Evidence from Archaeology and Genetics

**Figure 1:**
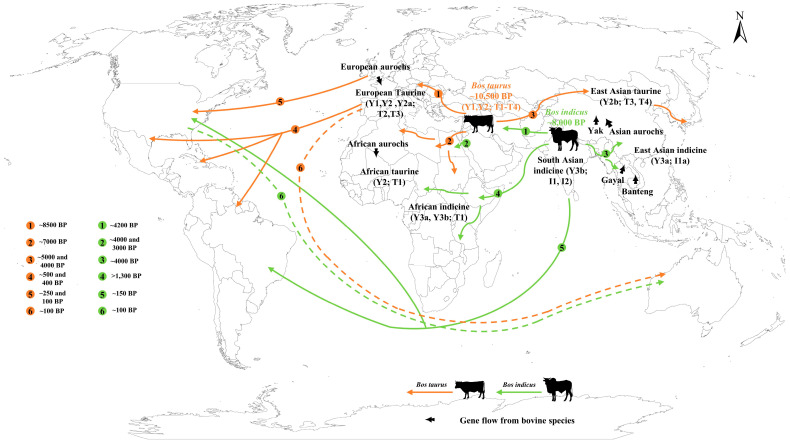
The domestication and dispersal route of taurine cattle and indicine cattle.

Domestic cattle possess at least two distinct centers of domestication (Figure 1). Approximately 10,500 years ago, in the Fertile Crescent of the Near East, European aurochs (*Bos primigenius primigenius*) were domesticated into taurine cattle (*Bos taurus*) [5]. Separately, over 8000 years ago, in the Indus Valley of South Asia, Indian aurochs (*Bos primigenius namadicus*) were domesticated into zebu cattle (*Bos indicus*) [6]. Molecular divergence of taurine and indicine (also referred to as zebu) lineages is estimated to have occurred at approximately 201,000–301,200 years ago [5,7,8,9,10], strongly supporting the two independent domestication events involving distinct aurochs subspecies. Both archaeological and genetic evidence consistently support separate domestication events, designating the Near East and the Indus Valley as primary centers for taurine and zebu cattle.

### 2.1. The Extinct Progenitor: Aurochs

The aurochs (*Bos primigenius*) was the direct progenitor of both humpless taurine and humped zebu cattle [4]. This bovine species once inhabited the temperate and subtropical biomes of Eurasia and North Africa during the Pleistocene and Holocene epochs. The species survived until the 17th century, and the last documented individual died in Poland in 1627 [4]. Early morphological comparative studies distinguished fossils of taurine cattle, zebu cattle, and their wild ancestors as separate species [11]. Aurochs have been classified into four distinct ancestries by both autosomal and mtDNA variation: European, South Asian, Southwest Asian or North African, and North Asian populations [12]. Aurochs from southern Europe diverged from central and northern European populations. During the Last Glacial Maximum, European aurochs contracted into southern Europe, specifically the Iberian, Italian, and Balkan peninsulas. Italy and the Fertile Crescent harbor the T haplogroup, dominant in modern domestic cattle, while central and northern European aurochs gave rise to the P haplogroup, which expanded from the Iberian Peninsula following the Last Ice Age [12,13]. At the onset of the Neolithic, European aurochs with different mtDNA haplogroups coexisted on the European and Southwest Asian continent: the P haplogroup persisted in Europe from the early Neolithic to the Bronze Age, the E haplogroup was distributed in Germany, and the T haplogroup was found in the Near East [14]. Notably, the P haplogroup is virtually absent in modern cattle, demonstrating that northern European aurochs provided a negligible contribution to the domestic gene pool, supporting the hypothesis of an exogenous origin for European domestic cattle [15,16]. Indian aurochs were widely distributed in the Indian subcontinent during the Pleistocene to Holocene [11]. In East Asia, ancient DNA from fossils in Northeast China has revealed a unique C haplogroup associated with East Asian aurochs [17,18,19]. Since the Last Glacial Maximum (LGM), gene flow has occurred between the western aurochs and Central Asian populations [20]. During the Holocene, the North Asian wild populations declined and ultimately became extinct in Northeast China as a result of climate deterioration and intensified human activity [19].

### 2.2. The near Eastern Center: Origin of Taurine Cattle

The domestication of cattle began with the capture of a limited number of individuals from wild aurochs populations in the Near East [21]. Subsequently, domesticated populations dispersed beyond their area of origin, with the ancestral strain of aurochs undergoing male-mediated genetic admixture [12]. Archaeological excavations in the Near East have provided the earliest irrefutable evidence of cattle domestication. Sites in the Middle Euphrates valley (10,800–10,300 years ago), the Tigris basin (~10,200 years ago), and the Northern Jordan Valley (during the 8th millennium) exhibit the initial morphological indicators of domestication, such as reduction in sexual dimorphism and body size [22,23,24]. By 8800 years ago, domestic cattle had expanded into western Anatolia and spread throughout southeastern Europe [23]. Additionally, by 7000 years ago, evidence of milk consumption was found in pottery from burial sites in both the Near East and southeastern Europe [25]. Mitochondrial DNA (mtDNA) provides the most robust evidence supporting the Near Eastern origin. The Near East encompasses mtDNA taurine lineages (T, T1, T2, T3, T5), exhibiting high genetic diversity [26,27]. Moving toward peripheral regions such as Europe and Africa, this genetic diversity gradually decreases: modern European cattle primarily carry the taurine T3 lineage, while the T1 lineage is predominantly distributed in Africa [26,27]. During the Late Bronze Age (~4200 years ago), widespread drought led to rapid and extensive genetic introgression of Indian cattle into domesticated cattle populations in the Southern Levant [28]. The introduction of zebu male individuals enhanced the herd’s survival capacity in arid environments.

### 2.3. The South Asian Center: Origin of Zebu Cattle

The domestication of zebu cattle represents an independent event involving the Indian aurochs. The archaeological site of Mehrgarh in Baluchistan serves as a pivotal reference for this process, dating to the mid-to-late Holocene (approximately 8000 years ago). Beginning in the 7th millennium, the proportion of domestic cattle bones among bovine remains increased rapidly [11]. Over 5000 years ago, domesticated zebu cattle had become widely distributed across the Indus and Ganges basins [6]. Zebu cattle genetically harbor the mtDNA I1 and I2 haplogroups. The diversity of I1 and I2 haplogroups in the northern Indian subcontinent is higher than in other parts of Asia [6]. On a finer geographic scale, samples from the Indus Valley show the highest diversity of the I1 haplogroup, surpassing that of southern India and the Ganges region [6]. The mitochondrial indicine I1 haplogroup spread east from northwestern India, with the I2 haplogroup sparsely distributed in India and southern China [7,29,30].

## 3. Geographic Lineages and Dispersal History of Modern Cattle

Following primary domestication, cattle dispersed outward from the Near East and Indus Valley, propelled by human migration, trade, and conquest. This dispersal was not a simple replacement of wild populations but a complex process involving introgressive hybridization with local wild aurochs and other *Bos* species. Modern cattle populations can be classified based on three genetic systems: mitochondrial, Y-chromosomal, and autosomal DNA. The autosomal genetic structure of modern cattle is primarily divided into six ancestries (Table 1): European taurine, East Asian taurine, African taurine, South Asian or Indian indicine, East Asian or Chinese indicine, and African indicine ancestry [9,10,31]. Phylogenetic patterns inferred from insertion and deletion markers are congruent with those obtained from SNPs [32]. As mentioned, the main mitochondrial lineages of modern cattle are the mtDNA taurine T lineage and the zebu I lineage [8]. The T lineage primarily includes T, T1, T2, T3 haplogroups, along with T3a or T4 haplogroups derived from T3 haplogroup, while T1 haplogroup differs by only two mutations in the control region from T3 haplogroup [5]. The T3 haplogroup is mainly distributed in Eurasia; the T2 haplogroup is found in Anatolia and the Middle East; the T1 haplogroup is mainly distributed in Africa; and T4 haplogroup is mainly distributed in East Asia [14,26,27,33]. Studies based on Y-chromosome microsatellites or SNP markers classify modern cattle paternal origins into taurine Y1, Y2 (Y2a and Y2b), and indicine Y3 (Y3a and Y3b) haplogroups [9,10]. The Y2 haplogroup is distributed across Eurasia and Africa, whiel Y2a sub-haplogroup is dominant in Eurasian cattle and Y2b sub-haplogroup is found having a higher proportion in Northeast Asian and Qinghai-Tibetan Plateau cattle [9,34]. The Y1 haplogroup is predominantly found in northern Europe [34]. Modern Indian cattle maintain a higher frequency of Y-chromosome indicine haplogroup compared to cattle in other regions [35]. The divergence of Y3a from Y3b, as well as sub-haplogroups within indicine mitogenomes, occurred approximately 23,100–24,800 years ago, while South Asian indicine ancestry diverged from East Asian and African indicine ancestry approximately 10,300–40,100 and 11,800 years ago, respectively [9,10]. The mtDNA I1 haplogroup and Y3b sub-haplogroup are widely distributed in India and other parts of the world, while Y3a sub-haplogroup predominates in southern China and Southeast Asia [6,9,36,37].

### 3.1. The Colonization of Europe

Following domestication in the Near East, cattle entered Europe from Anatolia around 8500 years ago, subsequently reaching southern Europe and spreading northward along the Danube over the next millennium [41,42]. The European continent in the early Neolithic was a region where European wild aurochs and domestic cattle coexisted. Despite this coexistence, the maternal lineages of European cattle remained overwhelmingly Near Eastern (T3), whereas male-mediated gene flow from local aurochs occurred [27,34]. Modern northern European cattle populations maintain a high frequency of Y1 haplogroup, directly reflecting the hybridization with male aurochs during the dispersal of domestic cattle into northern Europe [34]. In southern European populations, such as Italian cattle, small amounts of mitochondrial Q and R haplogroups are present [39,40]. The P, Q, and R haplogroups represent ancient branches parallel to the T lineage, indicating secondary introgression of European aurochs genes during the expansion of cattle populations into Europe [5,39,40]. The establishment of European cattle was also influenced by early African pastoralist maritime trade. The T1 haplogroup constitutes 5–30% of cattle in Mediterranean coastal areas, such as Spain and Greece, whereas it is absent in central and northern Europe [26]. By the sixteenth century, European cattle herds had become the primary settlers of the Americas and Oceania. Modern European cattle gave rise to the world’s most productive dairy and beef breeds. For example, the Simmental breed, which emerged in the Middle Ages in the Simme Valley of Switzerland, resulted from the crossbreeding of large German cattle with smaller Swiss indigenous cattle [43,44].

### 3.2. The African Mosaic

Approximately 7000 years ago, taurine cattle were distributed in southern Egypt and across the African continent, established by the T1 mtDNA lineage, which is now characteristic of African cattle [26,37,38,45]. Ancient Moroccan wild ox samples exhibit a closer kinship to the African taurine ancestry than to the European taurine ancestry, while African taurine cattle possess ~20% ancestry attributed to African aurochs, providing a genetic signature distinct from their European counterparts [46]. Zebu cattle were introduced 4000–3000 years ago, facilitated by maritime trade across the Indian Ocean and the Horn of Africa [4]. Significant influxes occurred during the Arab expansion (~700 AD), introducing male zebu cattle with the Y3b and Y3c sub-haplogroups, and again in the late 19th century, when cattle of the Y3a sub-haplogroup were introduced to restock herds devastated by the Rinderpest pandemic [36]. Notably, the indicine ancestry in African cattle does not entirely correspond with northern Indian zebu cattle, but shows similarities to Southeast Asian zebu cattle, which indicates exchanges between zebu cattle introduced to Africa and Southeast Asian herds in the expansion [46,47]. Zebu cattle spread westward from the Abyssinian region across the continent and penetrated south to Zambia, forming two major hybrid zones of taurine and zebu cattle in West and Southern Africa [48,49,50]. Consequently, most modern African breeds possess the mtDNA taurine T1 haplogroup but carry zebu Y-chromosomal indicine Y3 haplogroup. This pattern characterizes male-mediated gene flow, where zebu bulls were imported and crossed with local taurine cows to confer drought tolerance. Pure African taurine breeds are found exclusively in West Africa [51], largely due to their unique resistance to trypanosomosis, while Sanga breeds have been preserved through selective breeding preferences and geographic isolation [49]. As previously mentioned, African cattle (mtDNA T1, mixed with zebu lineage) were once introduced to the European Mediterranean region [26,52,53]. Subsequently, in the 15th century, southern European herds carrying African indicine ancestry were introduced to the Americas [52,53]. Breeds like the Texas Longhorn underwent continuous crossbreeding with zebu ancestry [54], facilitating the spread of African cattle in the Americas and globally. 

### 3.3. The East Asian Melting Pot

During the late Neolithic (4000–5000 years ago), taurine cattle were introduced to East Asia from West Asia, carrying T3 and the East Asian-specific T4 haplogroups [9,33,55,56,57]. They entered Mongolia and northern China [58], before spreading from Northeast China to Japan via the Korean Peninsula. Subsequently, the Yellow River Basin in China’s Central Plains region experienced at least two waves of cattle migration from the north. The first occurred from the late Neolithic to the Bronze Age, involving domestic cattle migrating from Western Eurasia that underwent approximately 10% genetic admixture with local wild cattle. The second wave took place from the late Bronze Age to the Iron Age, involving the expansion of Xinjiang-related ancestry into northern China [20]. Similarly, gene flow has been observed from East Asian aurochs into ancient and present-day Tibetan cattle [18]. European taurine ancestry entered after the Medieval period, further enriching the genetic diversity of modern East Asian cattle. Tibetan and Northeast Asian cattle breeds share an East Asian taurine ancestry component, and the Y2b sub-haplogroup was likely maintained by geographical barriers [9]. In contrast, Eurasian taurine ancestry and the Y2a sub-haplogroup predominated in cattle from Northwest China and south-central Europe [9]. 

Zebu cattle originated in India and reached Southeast and East Asia approximately 5500–2500 years ago [59]. Indochinese and Chinese cattle populations are dominant in the zebu ancestry, specifically Y-chromosomal sub-haplogroup Y3a and the maternal sub-haplogroup I1a [10,60,61]. Zebu cattle reached the Central Plains of China around 3500 years ago, forming a convergence zone of taurine and zebu cattle [55,62,63]. Zebu ancestry was widely distributed across northern China in the Medieval period [20]. A branch of the Indian zebu I1 haplogroup formed the Chinese zebu I1a sub-haplogroup, while the genome-wide nucleotide diversity was higher in East Asian indicine cattle than in South Asian indicine cattle [10]. Populations from Guangxi and Fujian in southern East Asia reached Southeast Asia about 4000 years ago [64]. A previous study also reported an expansion of cattle carrying I1a sub-haplogroup around 3730 years ago [10], indicating a marked increase in zebu cattle dispersal and exchange frequency beginning approximately 4000 years ago. While ancient DNA evidence remains sparse, the formation of southern Chinese indicine cattle represents a critical frontier for future genomic research.

The rapid adaptation of East Asian cattle to hot and humid environments and high-altitude environments was promoted by localized introgression from other bovine species [65,66]. The introgression proportions of banteng (*Bos javanicus*), gaur (*Bos gaurus*), and yak (*Bos mutus*) ancestries into East Asian indicine or Tibetan taurine cattle ranged from 1.13% to 10.21%, 2.06% to 9.98%, and 0.05% to 2.94%, respectively [9,10]. In addition, kouprey-like and gayal-like ancestries accounted for 3.2% and 1.4% in Chinese indicine genomes [67].

### 3.4. The History of Introduction to the Americas and Oceania

In the late 15th and 16th centuries, European cattle, especially southern stock, reached the Americas with Spanish colonizers. Historical records indicate that during Columbus’s second voyage in 1493, European cattle were introduced from the Iberian Peninsula to the Caribbean islands [4]. They subsequently spread throughout the Americas with Spanish colonists during the 16th century: transported from the Caribbean to Mexico in 1521, reaching Colombia by 1524, and continuing to spread southward [4]. By 1540, they had expanded north to Texas, giving rise to the Texas Longhorn breed [54]. From the 17th to 19th centuries, specialized dairy and beef breeds were introduced from Europe. To enhance productivity, particularly heat tolerance and disease resistance, zebu breeds such as Kankrej, Ongole, and Gir were introduced from 1813 onwards, later yielding the Brahman favoured in tropical regions and indicine × taurine crossbreds such as the Beefmaster [59,68]. In Australia, British cattle breeds including Holstein, Shorthorn, Hereford, and Angus were introduced from the 19th century [4]. Later, zebu cattle were similarly incorporated to enhance environmental adaptability. Following the severe threat posed by cattle tick infestations to tropical Queensland herds in 1896, zebu cattle from India and the United States were introduced for crossbreeding to confer tick resistance [4]. In New Zealand, only European cattle were introduced, primarily dairy breeds originating from Britain and the Netherlands [4].

Modern domestic cattle in the United States and Australia trace their lineages to two primary ancestral groups: European cattle and Indian humped cattle. Brazilian and American breeds such as Creole and Nellore share similar genetic backgrounds with southern European cattle. They carry the T3 and T1 mitochondrial haplogroups and exhibit indicine ancestry [54,69,70,71,72,73,74,75]. Furthermore, ancient DNA studies show that cattle introduced to the Americas in the early 17th century came from diverse regions. These included European cattle from the Iberian Peninsula (haplogroup T3) and African cattle (sub-haplogroup T1b) brought by the slave trade [76]. This historical introduction resulted in direct African genetic contributions to Creole cattle from Brazil and Colombia [72]. This diverse genetic background contributed to their remarkable ability to adapt to the harsh, semi-arid environments of the American Southwest and the tropics of Latin America. Beef production exhibits a significant geographic concentration, primarily within North and South America, with Brazil and the United States standing out as major global players in both production and consumption [77,78,79]. Nellore is the main beef cattle breed produced in Brazil, and more than 80% of Brazil’s beef cattle population comprises purebred or hybrid Nellore cattle [79]. In the United States, the establishment of the “Certified Angus Beef” brand in 1978 further focused the breeding objectives for intramuscular fat content, achieving standardized and premium product quality [80].

## 4. Environmental Adaptive Evolution of Cattle

**Figure 2:**
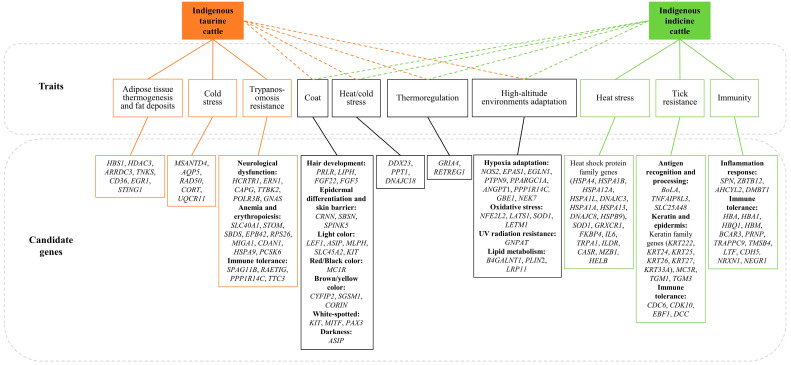
Candidate genes associated with environmental adaptations in indigenous taurine and indicine cattle. Dashed lines indicate selection signals specific to each group or their crossbred populations.

Natural selection has reshaped the cattle genome, enabling cattle to thrive in diverse geographical and climatic environments. Recent studies employing whole-genome sequencing, transcriptomics, genome-wide association analysis, and selection signal detection have allowed the precise identification of the specific molecular mechanisms underlying environmental adaptations, such as thermal tolerance, disease resistance, and high-altitude adaptation (Figure 2 and Table 2). The unique genomic characteristics and adaptive genomic regions of indigenous breeds can support future conservation and breeding efforts aimed at maintaining stress resilience under environmental changes.

### 4.1. Thermal Adaptation

Cattle breeds in high-latitude regions, such as the Yakut of Siberia and Yanbian cattle of China, face extreme cold stress. These cattle in cold regions typically possess thicker fur and subcutaneous fat layers to insulate against cold and reduce heat loss. In the Siberian region where winter temperatures drop to −50 °C, genome scanning has identified candidate genes potentially associated with cold adaptation in Russian cattle breeds. These genes include those involved in temperature sensation (*RETREG1*), cold-regulated water channels (*AQP5*), adipose tissue and thermogenesis regulation (*TNKS*, *ARRDC3*, *HDAC3*), and DNA repair (*RAD50*) [92]. A GWAS analysis in Siberian cold-tolerant breeds (Hereford and Kazakh Whiteheaded) identified candidate genes *MSANTD4* and *GRIA4*, related to cold shock response and thermoregulation, respectively [91]. Additionally, genes related to cold/heat stress response (*DDX23*, *PPT1*) and thermogenesis (*GRIA4*, *HBS1*) were found to be under selection [90]. *GRIA4* has been reported as a candidate gene for heat tolerance in Australian Holsteins, enriched in the glutamatergic synapse pathway [106], suggesting that *GRIA4* mediates response to both cold and heat stress through thermoregulation. *HBS1*, which is associated with the GO term “regulation of lipid transport”, was found to be downregulated in Yakutian cattle [107]. *DDX23*, a heat shock gene, showed up-regulation in Sahiwal peripheral blood mononuclear cells from tropical regions relative to Ladakhi cattle from hypobaric hypoxia regions [108,109]. In Yanbian cattle of northeastern China, genes related to cold stress (*CORT*), hair follicle and hair growth (*FGF5*), and lipid metabolism (*CD36*) were selected [93]. In another study, genes related to adipose tissue thermogenesis (*EGR1*, *STING1*), cold stress (*DNAJC18*, which is also under selection in African zebu cattle [99]), and oxidative phosphorylation (*UQCR11*) were identified as candidate genes in northern Chinese cattle [94]. Furthermore, compared with commercial dairy breeds, genes related to body size (*TIGAR*, *CCND2*, *CSMD3*) underwent strong selection in Fjällnära of northern Sweden, indicating that small body size is an adaptive strategy for food shortages in cold environments [110].

In hot environments, animals typically regulate their body temperature through physiological and behavioral changes such as increasing water intake, reducing food consumption, seeking shade, and altering breathing rates. Tropical zebu breeds possess morphological features such as dewlaps, sparse coats, developed sweat glands, and light-colored hair to facilitate heat dissipation [111]. The heat shock protein (HSP) family protects cells from thermal damage and prevents protein denaturation. Genes involved in heat tolerance have been identified in Indian and African indicine cattle populations, such as heat shock protein genes (*HSPA1B*, *HSPA12A*, *DNAJC18* and *DNAJC8*), *GRXCR1*, *FKBP4*, *HSPA1L*, *IL6*, and *HELB* [81,100,112,113]. *HSPA1A* and *HSPA1B* expression vary seasonally in zebu cattle, being higher in summer than in spring [114]. *HSP70* expression was higher in Sahiwal than in Holstein Friesian [115], suggesting that zebu cattle may have greater heat tolerance. In another Indian breed, Tharparkar, a SNP (g.149G > T) in *HSP70* was associated with thermoregulatory ability under hot conditions [116]. Additionally, the expression of *TLR2/4* and *IL2/6* in blood was upregulated under both short-term and long-term heat stress [117]. Compared with commercial taurine cattle, the superoxide dismutase gene *SOD1* and prolactin releasing hormone gene *PRLH* were under selection in African indicine cattle populations [82]. Indicine cattle in East Asia and Southeast Asia live in more humid environments than those in South Asia. Genes associated with heat stress response (*DNAJC3*, *HSPA1A*) and inflammatory response (*CD53*, *ZBTB12*, *AHCYL2*) were under selection in Hainan cattle [102]. Genomic signals of recent selection and adaptive introgression from banteng and gaur into East Asian indicine cattle have been identified. These candidate genes are involved in multiple functions: heat-sensing proteins (*TRPA1*), paracellular water transport and urine concentration regulation (*ILDR*), Ca^2+^ homeostasis (*CASR*), and resistance to tick-borne diseases (*HBA*, *HBA1*, *HBQ1*, *HBM*) [10]. Recent pangenome studies have highlighted the importance of structural variants in adaptation. For example, a 108 bp insertion (INS) in *SPN* generates an additional 36-amino-acid repeat sequence within the extracellular domain of CD43 [101]. This insertion correlates with an increased number of O-linked glycosylation sites and confers resistance to *M. tuberculosis* in Hainan cattle [101].

Domestic cattle in tropical regions have short coats and light-colored fur. Some coat-related genes, such as *PRLR*, have undergone artificial selection. The *PRLR* gene (prolactin receptor), which is linked to the short, sleek coat phenotype, was found to be under selection in Latin American Criollo cattle [73]. These cattle were introduced to Africa from South America during the colonial trade period [118]. Selection for *PRLR* enhances heat tolerance by modifying hair coat length and structure. In a comparison of light- and dark-coated South Asian indicine cattle breeds, pigmentation-related genes (*LEF1*, *ASIP*) were under selection, as well as hair development genes (*LIPH*, *FGF22*) [10]. Specific SVs in *CRNN*, *SBSN* and *SPINK5* were also identified in Hainan and Mongolian cattle, affecting skin barrier function and heat adaptation (hair follicle density, sweat gland area) [101]. In summary, thermal adaptation involves a complex network of genes regulating thermogenesis, heat and cold stress response, lipid metabolism and immunity. However, research based on SNP-phenotype associations still lacks functional validation to fully elucidate the underlying molecular mechanisms. 

### 4.2. Parasitic Disease Resistance

In tropical regions, pathogens such as trypanosome parasites (transmitted by tsetse flies) and vectors like ticks exert immense selective pressure. Consequently, cattle have evolved complex defense mechanisms, ranging from physical barriers to immune responses. The skin and mucosal barriers serve as the first line of defense. In African cattle, genomic selection signatures have been identified in genes involved in antigen response (*BoLA*, *SLC25A48*), keratin formation (*KRT33A*, *KRT222*, *KRT24*, *KRT25*, *KRT26*, *KRT27*, *TGM1*, *TGM3*), light coat color (*MLPH*), and sebum secretion (*MC5R*) [81,82,99,103]. Notably, the keratin genes *KRT5* and *KRT14* are highly expressed in cattle populations exhibiting high tick tolerance [119]. Mechanistically, a keratinized epidermis may trigger an inflammatory response upon early tick infestation, while a light-colored coat and sebum secretion likely reduce tick attachment. Regarding intestinal mucosal defense, the *DMBT1* gene, associated with resistance to intestinal parasites, has also undergone selection [81]. Similarly, in southern Chinese cattle, genes related to tick and bacterial resistance (*LTF*, *TGM3*, *BCAR3*) show evidence of being under selection [105].

Sub-Saharan Africa is a high-incidence region for bovine trypanosomosis, with tsetse flies serving as the primary vector [120,121]. Cattle employ behavioral strategies to mitigate infection. Individuals exhibit increased fly-repelling behavior when biting fly density is high, such as ear twitching, head shaking, and tail swishing [122]. Studies suggest that individuals displaying frequent repelling behavior are bitten by fewer flies [122]. Physiologically, African cattle maintain biological function post-infection by regulating feeding behavior, iron homeostasis, and erythropoiesis. In the N’Dama breed, known for its trypanotolerance, the *HCRTR1* gene (encoding hypocretin receptor 1) and anemia-related genes (*SLC40A1*, *STOM*, *SBDS*, *EPB42*, *RPS26*) were found to be under strong selection [82]. In Sheko, another trypanotolerant breed, 15 genes related to anemia, immune tolerance, and neurological dysfunction (*MIGA1*, *CDAN1*, *HSPA9*, *PCSK6*, *ERN1*, *CAPG*) showed signatures of selection [104]. Comparative transcriptomic analysis between N’Dama and the trypanosusceptible Boran breed has revealed significant differences in response to infection. Differential expression was observed in immune-related genes, specifically those encoding antimicrobial peptides (*LEAP2*, *CATHL3*, *DEFB4A*, *S100A7*) and cytokines (*CCL20*, *CXCL11*, *CXCL13*, *CXCL16*, *CXCL17*, *IL33*, *TNFSF13B*), as well as genes regulating coagulation and iron homeostasis (*SLC40A1*, *SLC11A1*) [123]. These findings support the hypothesis that the dual capacity to control both parasitaemia and the anemia resulting from the innate immune response is key to trypanotolerance [123].

### 4.3. High-Altitude Adaptation

The Qinghai-Tibetan Plateau has an average altitude exceeding 4000 m, characterized by extreme environmental conditions including strong ultraviolet radiation, hypoxia, low temperatures, and limited forage availability. Cattle inhabiting this region have evolved distinctive physiological strategies and have acquired adaptive alleles through ancient hybridization with yaks. In Zhangmu cattle, hypoxia-adaptive genes at the core of the hypoxia-inducible factor pathway, including *EPAS1* and *EGLN1*, as well as the nitric oxide synthase gene *NOS2*, were shown to be introgressed from yaks [97]. A structural variation analysis further revealed that 7293 SVs in Tibetan cattle originated from yak introgression. These variants included an upstream regulatory variant of *EGLN1* and intronic deletions in *PPP1R14C* and *NFE2L2*, which are associated with angiogenesis and glucose transporter 1 upregulation, respectively [89]. Lyu et al. [98] reported that three yak-derived SNPs in the promoter region of *EGLN1* may enhance hypoxia tolerance. Introgression signals were also detected for genes involved in cold adaptation (*LRP11*), DNA damage repair (*LATS1*), and ultraviolet radiation resistance (*GNPAT*) [98]. Compared with low-altitude breeds such as Angus, Wu et al. [96] identified strong selection signals in *ANGPT1* and *PPARGC1A*, two genes functionally related to hypoxia adaptation. *ANGPT1* belongs to the angiopoietin family, while *PPARGC1A* is associated with muscle fiber type specification; both genes are fundamental to improved oxygen delivery under high-altitude conditions. Liu et al. [97] screened a panel of candidate genes associated with high-altitude adaptation in Tibetan cattle, including the protein tyrosine phosphatase family member *PTPN9*, immune regulatory gene *IL6*, and lipid metabolism-related genes *B4GALNT1* and *PLIN2*. In the Ethiopian highland breed Gojjam, which resides at altitudes above 4000 m, genes involved in the hypoxia-inducible factor-1 signaling pathway (*GBE1*, *NEK7*) and oxidative stress resistance (*SOD1*) exhibited strong signatures of selection [99]. Tibetan taurine cattle displayed significant selection signals in *LETM1*, *TXNRD2*, and *STUB1*, which may promote hypoxia adaptation by modulating mitochondrial function and HIF-1α stability [95]. In Apeijiaza cattle and Shigatse Humped cattle, both of which carry indicine ancestry, genes associated with cardiovascular function (*NOXA1*, *RUVBL1*, *SLC4A3*) underwent intense selection [95]. Structural variation analyses further identified genes involved in erythropoiesis and angiogenesis (*SSH2*, *VGLL4*, *PLCB1*, *HPSE2*, *HPSE*) and energy metabolism (*SORD*, *NDUFB6*, *SARDH*, *ADIPOQ*) [89]. Additionally, genes related to small body size (*HMGA2*, *NCAPG*) and thyroid hormone-mediated energy metabolism (*DUOXA2*) showed clear signatures of selection in high-altitude cattle populations, constituting a specialized adaptive response to low-energy nutritional environments [98]. Collectively, these candidate genes participate in hypoxia response, erythropoiesis, angiogenesis, and metabolic regulation, and jointly facilitate the adaptive evolution of cattle to extremely high-altitude environments.

The Qinghai-Tibetan Plateau has an average altitude exceeding 4000 m, characterized by extreme environmental conditions including strong ultraviolet radiation, hypoxia, low temperatures, and limited forage availability. Cattle inhabiting this region have evolved distinctive physiological strategies and have acquired adaptive alleles through ancient hybridization with yaks. In Zhangmu cattle, hypoxia-adaptive genes at the core of the hypoxia-inducible factor pathway, including *EPAS1* and *EGLN1*, as well as the nitric oxide synthase gene *NOS2*, were shown to be introgressed from yaks [97]. A structural variation analysis further revealed that 7293 SVs in Tibetan cattle originated from yak introgression. These variants included an upstream regulatory variant of *EGLN1* and intronic deletions in *PPP1R14C* and *NFE2L2*, which are associated with angiogenesis and glucose transporter 1 upregulation, respectively [89]. Lyu et al. [98] reported that three yak-derived SNPs in the promoter region of *EGLN1* may enhance hypoxia tolerance. Introgression signals were also detected for genes involved in cold adaptation (*LRP11*), DNA damage repair (*LATS1*), and ultraviolet radiation resistance (*GNPAT*) [98]. Compared with low-altitude breeds such as Angus, Wu et al. [96] identified strong selection signals in *ANGPT1* and *PPARGC1A*, two genes functionally related to hypoxia adaptation. *ANGPT1* belongs to the angiopoietin family, while *PPARGC1A* is associated with muscle fiber type specification; both genes are fundamental to improved oxygen delivery under high-altitude conditions. Liu et al. [97] screened a panel of candidate genes associated with high-altitude adaptation in Tibetan cattle, including the protein tyrosine phosphatase family member *PTPN9*, immune regulatory gene *IL6*, and lipid metabolism-related genes *B4GALNT1* and *PLIN2*. In the Ethiopian highland breed Gojjam, which resides at altitudes above 4000 m, genes involved in hypoxia-inducible factor-1 signaling pathway (*GBE1*, *NEK7*) and oxidative stress resistance (*SOD1*) were found to be under strong selection [99]. Tibetan taurine cattle displayed significant selection signals in *LETM1*, *TXNRD2*, and *STUB1*, which may promote hypoxia adaptation by modulating mitochondrial function and HIF-1α stability [95]. In Apeijiaza cattle and Shigatse Humped cattle, both of which carry indicine ancestry, genes associated with cardiovascular function (*NOXA1*, *RUVBL1*, *SLC4A3*) underwent intense selection [95]. Structural variation analyses further identified genes involved in erythropoiesis and angiogenesis (*SSH2*, *VGLL4*, *PLCB1*, *HPSE2*, *HPSE*) and energy metabolism (*SORD*, *NDUFB6*, *SARDH*, *ADIPOQ*) [89]. Additionally, genes related to small body size (*HMGA2*, *NCAPG*) and thyroid hormone-mediated energy metabolism (*DUOXA2*) showed clear signatures of selection in high-altitude cattle populations, constituting a specialized adaptive response to low-energy nutritional environments [98]. Collectively, these candidate genes participate in hypoxia response, erythropoiesis, angiogenesis, and metabolic regulation, and jointly facilitate the adaptive evolution of cattle to extremely high-altitude environments.

## 5. Artificial Selection and Breed Diversification

**Figure 3:**
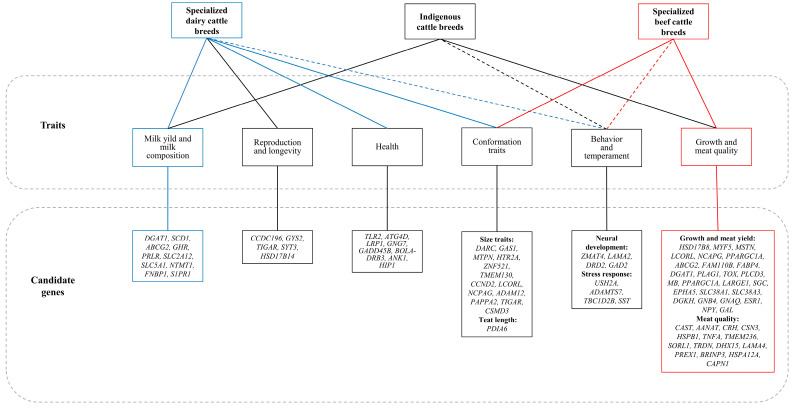
Candidate genes associated with economic traits in specialized dairy and beef cattle breeds. Dashed lines indicate unspecified group assignments.

During domestication, cattle underwent changes in temperament, behavior, coat color, horn morphology, and reproductive cycles. These phenotypic changes are usually closely related to biological processes such as the nervous system, growth metabolism, and immunity [124,125]. Artificial selection leaves signatures in the exon regions of the genome [126]. Through high-intensity breeding, modern cattle have undergone significant changes in lactation, meat performance, and health traits [127] (Figure 3).

### 5.1. Temperament Selection

Docility is a hallmark behavioral trait distinguishing domesticated animals from their wild counterparts, and its intensification during domestication is tightly correlated with a substantial reduction in relative brain volume. For instance, domestic cattle exhibit a 25.6% reduction in brain volume compared to wild oxen, with docile dairy breeds showing twice the magnitude of this reduction relative to aggressive fighting breeds [128]. Recent genomic and transcriptomic studies have begun to unravel the genetic architecture underlying these temperament differences. In dual-purpose Simmental cattle, four candidate genes have been linked to milking response: *ZMAT4* (neural development), *USH2A*, *ADAMTS7*, and *TBC1D2B* (stress response) [129]. Among these, *ADAMTS7* is associated with muscle tone and joint sensitivity, while *USH2A* modulates sensory perception through auditory and tactile pathways [129]. Complementing this, a meta-analysis by Ruiz et al. [130] identified the stress-responsive gene *SST* and members of the synaptic function-related Kelch family as key regulators of cattle temperament. Transcriptome profiling of the prefrontal cortex further highlights interbreed differences. Compared with Wagyu cattle, the highly aggressive Lidia breed shows upregulated expression of genes enriched in pathways linked to abnormal aggression and neurophysiological disorders, including *LAMA2*, *DRD2*, and *GAD2* [131]. Behaviorally, temperament traits such as flight speed in beef cattle are known to be heritable [132,133], directly impacting an animal’s response to human handling during milking, transport, and calving. These traits consequently influence management efficiency, animal welfare, and overall production performance [134]. Despite these advances, the biological mechanisms governing cattle temperament remain highly complex, and no gene with a major effect on behavioral phenotypes has been identified to date [135]. Future research should focus on in-depth investigation of behavior-related loci, coupled with refined behavioral phenotyping and improved data acquisition systems, to elucidate the genetic basis linking stress behavior to production efficiency. Ultimately, incorporating temperament traits into selective breeding programs to favor low stress responsiveness will not only optimize modern breeding strategies but also enhance animal welfare [136,137].

### 5.2. Coat Color Selection

Cattle exhibit diverse coat colors. Some researchers consider coat color an environmental adaptation: light-colored coats reduce radiation absorption and tick infestation, facilitating adaptation to tropical and subtropical environments [138]. However, preference for particular coat colors represents a non-negligible artificial selection pressure. Red (yellow) and black (brown) coat colors are determined by the ratio of eumelanin to pheomelanin, which is regulated by variations in melanogenesis pathway-related genes *ASIP*, *MC1R*, and *KIT*. *MC1R* mutations generate the red coat color phenotype in Ankole and Evolèner cattle populations [82,139]. Compared to red-brown Sahiwal, black and white Karan Fries carry nucleotide mutations in *MC1R* [140]. *MC1R* is also a candidate gene for black Zhoushan cattle and for black-and-brown coat color in Nguni cattle [85,86]. Structural variations in *ASIP* are prevalent in zebu cattle populations (45.9%) [141]. Nellore cattle are predominantly white, but localized gray-to-black hair on the head, neck, hump, and knees in bulls is related to *ASIP* variation [87]. In brown Guanling cattle, skin *ASIP* expression is substantially higher than in black Angus cattle, whereas melanin content shows the opposite trend [142]. *KIT*, *MITF* and *PAX3* are key regulators of melanocyte development, migration, and differentiation in cattle with white spotting patterns [88]. Long-range and intronic cis-regulatory variants in *KIT* and *MITF* determine spotting traits in Holstein-Friesian and other cattle breeds [143]. A 2 Mb heterozygous inversion and two translocations harboring genes including *KIT* were associated with the gray coat phenotype in Tibetan cattle, and the inversion haplotype segment originated from South Asian indicine cattle [89]. *KIT* is also related to color-sidedness and has been selected in Ankole with white spotting [82], although indicative SNPs for similar phenotypes in Bali and Nguni cattle are not directly linked to *KIT* [144]. *MAPK10*, *EFNA5*, *PPP2R3C*, and *PAK1* are candidate genes for the white forehead pattern in Nguni, possibly acting synergistically via MAPK, adrenergic, and Wnt pathways to affect melanin synthesis [145]. GWAS has shown that *CYFIP2* and *SGSM1* are closely related to coat color in Sumatran native cattle (black, brown, white) [84], while *CORIN* is a candidate for coat color in Leiqiong cattle (black, yellow) [83]. Haplotype analyses indicate that the yellow coat phenotype in Leiqiong cattle originates from Indian indicine ancestry, whereas the black coat phenotype emerged through introgression from kouprey and artificial hybridization with Wagyu cattle [83]. Evidently, cattle coat color is controlled by multiple genes and varies substantially among breeds.

### 5.3. Selection for Lactation Traits

Currently, the three primary dairy cattle breeds raised in major global dairy regions are Holstein, Jersey, and Brown Swiss, together with their crossbreeds [146]. Meanwhile, both total and per-animal milk production levels continue to increase [146]. Evolutionary analysis has shown that milk and mammary gland genes are more conserved than other genes across mammals [147]. Genomic studies have revealed that lactation-related genes, including *DGAT1*, *SCD1*, *ABCG2*, *GHR*, and *PR*, are strongly associated with milk yield and milk composition [148]. Non-synonymous SNPs in *DGAT1* K232A and *SCD1* A293V mainly affect milk fat content and fatty acid composition [149,150]. *DGAT1* encodes a microsomal enzyme that catalyzes triglyceride synthesis and serves as a key rate-limiting step for milk fat production [151,152]. Individuals carrying the lysine (K) allele exhibit a higher milk fat percentage in both Holstein and other indigenous dairy cattle breeds, whereas those with the alanine (A) allele show higher milk protein content and total milk yield [151,152,153,154]. Haplotype analysis has shown that taurine breeds carry a high frequency of the *DGAT1* A haplotype, while zebu breeds possess a high proportion of the K haplotype [154,155]. Using the XP-EHH method, Iso-Touru et al. [156] detected strong selection signals in *GHR* and *ABCG2* in Finnish dairy cattle. A GWAS revealed that *CTNNA3* affects milk protein concentration in first-lactation Holstein cows [157]. The genes *NTMT1*, *FNBP1*, and *S1PR1* have been reported as candidate genes influencing lifetime productivity in Holstein cows [158].

In addition to milk production traits, recent studies have identified genes associated with body conformation, reproduction, and disease resistance in dairy cattle breeds. By combining conformation-related genes with reported quantitative trait locus (QTL) regions, Wu et al. [159] identified *DARC*, *GAS1*, *MTPN*, *HTR2A*, *ZNF521*, *PDIA6*, and *TMEM130* as candidate genes for body capacity and depth, chest width, foot angle, angularity, rear leg side view, teat length, and body size, respectively, in the Chinese Holstein population. By integrating multiple expression datasets, allele frequency data from fertility-selected cows, and a GWAS for calving interval, numerous candidate genes strongly associated with dairy cow fertility have been identified, such as *CCDC196*, *GYS2*, *TIGAR*, *SYT3*, and *HSD17B14* [160]. Longevity is a key determinant of profitability in dairy systems, and studies have identified differences in health and productivity between herds with different longevity levels [161,162]. Sustainable and healthy cattle farming has received increasing attention, and several studies have reported candidate genes associated with disease resistance. *TLR2*, a key pattern recognition receptor for mycobacterial antigens, contributes to enhanced innate immune signaling and stronger anti-*Mycobacterium bovis* responses in Brown Swiss cattle [163]. In the Holstein population, candidate genes involved in resistance to bovine paratuberculosis have been identified using single-step genomic evaluation, including *GNG7*, *GADD45B*, *BOLA-DRB3*, *ANK1*, *HIP1*, the autophagy-related gene *ATG4D*, and the inflammatory response-related gene *LRP1* [164].

### 5.4. Selection for Meat Traits

Beef breeding programs commonly focus on carcass weight (CW), eye muscle area (EMA), yearling weight (YW), backfat thickness (BFT), and marbling score (MS). Genes such as *HSD17B8*, *HSPA12A*, *CAPN1*, and *MSTN* are candidates for growth and meat quality traits. Polymorphisms in *HSD17B8* affect body weight and average daily gain in Nanyang and Jiaxian cattle, and have also been associated with carcass weight and backfat thickness in specialized beef breeds such as Angus and Simmental [165]. *CCND2*, *LCORL*, *NCAPG*, *ADAM12*, and *PAPPA2* have been reported to play an important role in genetic variation in body stature in Belgian Blue cattle and multiple other species [166]. *MYF5*, *CAST*, and *MSTN* are associated with fattening traits in Brown Swiss and Holstein populations [167]. *MYF5*, which regulates skeletal muscle differentiation, has been identified as a candidate gene for carcass size and quality in Qinchuan cattle [168]. The myostatin gene *MSTN* regulates skeletal muscle and adipose tissue development, whereas the calpastatin gene *CAST* is associated with meat quality traits [169]. In Hanwoo, genomic regions under selection for carcass and growth traits are mainly located on chromosomes 6 and 14, including candidate genes *LCORL*, *NCAPG*, *PPARGC1A*, *ABCG2*, *FAM110B*, *FABP4*, *DGAT1*, *PLAG1*, and *TOX* [170,171]. Meat production is a major focus for the commercial improvement of Chinese indigenous cattle breeds. Selected lines of Qinchuan cattle (QNS) show strong selection in growth and meat quality genes (*PLCD3*, *MB*, *PPARGC1A*) compared with the original population (QCC) [172]. In Zaosheng cattle from Northwest China, *LARGE1*, *SGCZ*, and *EPHA5* have shown evidence of selection. *LARGE1* and *SGCZ* regulate muscle cell homeostasis, while *EPHA5* mediates satellite cell proliferation [173]. A transcriptome study of pituitaries from fast-growing Yunling cattle and slow-growing Leiqiong cattle identified candidate genes involved in growth hormone regulation, including *SLC38A1*, *SLC38A3*, *DGKH*, *GNB4*, *GNAQ*, *ESR1*, *NPY*, and *GAL* [174]. Meat quality is also influenced by nutritional and management conditions [175], and a number of genes associated with meat quality and nutritional traits have been identified. Sevane et al. [176] reported that *CAST* affects fat score; *HSPB1* influences the ratios of lauric acid (12:0) and DHA (22:6 n-3); *TNFA* affects lightness (L*); and *AANAT*, *CRH*, *CSN3*, *HSPB1*, and *TNFA* are associated with fatty acid composition. Xia et al. [177] identified genes involved in meat quality traits in Simmental, including fat color (*TMEM236*), meat color (*SORL1*, *TRDN*), marbling (*S100A10*, *AP2S1*), longissimus dorsi (*KCTD16*, *LOC506594*), and shear force (*DHX15*, *LAMA4*, *PREX1*, *BRINP3*). Comparative studies of Chinese indigenous and commercial beef cattle have revealed differential expression of the tenderness-related genes *HSPA12A* and *CAPN1* in the longissimus dorsi of Wenshan cattle and Simmental [178].

Milk and meat traits are closely linked to lactation and skeletal muscle growth. With the development of single-cell sequencing, cell-level spatiotemporal metabolic and regulatory analyses show great potential for identifying molecular markers for commercial breeding and supporting precise interventions in livestock production systems. It is important to recognize that intensive directional selection has greatly improved milk yield in dairy cattle and meat production in beef cattle. However, such intensive selection has also been accompanied by a reduction in genetic diversity within indigenous cattle populations, which may erode the adaptive genomic background and disease resistance of local breeds. Future research and breeding strategies should therefore balance the improvement of production performance with the conservation of genetic diversity.

## 6. The Pangenome Era: Unlocking Hidden Diversity and Missing Heritability

Pangenomes can be categorized into linear pangenomes and graph pangenomes, which employ distinct construction methods [179]. Traditional genomic studies have heavily relied on a single linear reference genome [180], such as ARS-UCD1.2, which is derived from an individual of a specific taurine breed, Hereford [181] (Table 3). While invaluable, this linear model fundamentally fails to capture the full genetic diversity, such as SVs and presence/absence variations (PAVs) [182], particularly in non-reference populations like *Bos indicus* [180]. These hidden variants explain a substantial fraction of the missing heritability in complex traits, such as disease resistance and adaptation, that GWAS based on linear reference genomes have failed to detect. The linear model inherently obscures sequences present in other individuals but absent in the reference, thereby introducing significant reference bias in variant calling and trait association [180]. Technologically, the field is moving from linear pangenomes to graph pangenomes. Graph pangenomes, facilitated by third-generation long-read sequencing, effectively resolve highly repetitive regions and centromeres [183]. Unlike linear concatenations, graph pangenomes represent genomes as network structures (nodes and edges), allowing for the simultaneous storage of multiple haplotypes and complex nested variations [183,184]. Recent efforts to construct pangenomes for Chinese and Indian indicine cattle have uncovered megabases of novel sequences. For instance, Dai et al. [67] identified 148.5 Mb of novel sequences in Chinese indicine cattle, which are enriched in gene families related to immune response and sensory perception, likely resulting from introgression from wild relatives such as banteng and gaur. Similarly, Azam et al. [185,186] identified 7.5 Mb of high-confidence non-reference unique insertions (NUIs) and 41Mb of non-reference novel sequences in Indian cattle. These insertions are enriched in immune-related gene clusters, further highlighting that indicine immune systems possess a genomic repertoire distinct from the taurine reference genome [186].

Beyond simple novel insertions, SVs including large deletions, inversions, and copy number variations (CNVs), are now recognized as major drivers of phenotypic variation, often exerting stronger effects on gene expression than SNPs by altering gene dosage or regulatory elements. Current pangenome analyses have resolved complex SVs that were previously “invisible” to short-read sequencing. A striking example is the complex SV situated 66 kb upstream of the *KIT* gene: a variable number of tandemly duplicated 14.3 kb repeats has been identified as the causal mutation for the white-head phenotype, a trait whose heritability was previously difficult to pinpoint using standard SNP arrays [187]. Furthermore, precise characterization of SVs has linked a 108 bp insertion in *SPN* to *M. tuberculosis* resistance and associated specific upstream variants of *EGLN1* to high-altitude adaptation [89,101].

**Table 3:** Chromosome-level genome assemblies for *Bos* species.

Species	GenBank Accession	Breeds	Assembled Length/Gb	Contig N50	Sequencing Technology	References
*Bos taurus*	GCA_002263795.4	Hereford	2.8	26.4 Mb	PacBio; Illumina NextSeq 500; Illumina HiSeq; Ilumina GAll	USDA ARS
	GCA_947034695.1	Charolais	3.2	84.1 Mb	PacBio; Hi-C; 10× Chromium	INRAE
	GCA_028973685.2	Hanwoo	3.1	64.7 Mb	PacBio	[188]
	GCA_021234555.1	Jersey	2.6	50.6 Mb	PacBio	USDA ARS
	GCA_034097375.1	Yunling cattle	3.1	36 Mb	PacBio	Yunnan Agricultural University
	GCA_905123885.1	African N’Dama	2.9	18.7 Mb	PacBio; Illumina	[189]
	GCA_905123515.1	African Ankole	2.8	11.1 Mb	PacBio; Illumina	[189]
	GCA_021347905.1	Holstein-Friesian	2.7	8.7 Mb	PacBio; Illumina	[188]
	GCA_000003205.6	Hereford	2.7	276.3 Mb	Sanger; PacBio RS Il	Cattle Genome Sequencing International Consortium
	GCA_000003055.5	Hereford	2.7	97 Mb	Sanger	[190]
	GCA_003369685.2	Angus	2.6	102.8 Mb	PacBio; Hi-C; Illumina NextSeq; Sequel	[191]
	GCA_049634565.1	Hanwoo	3.1	90.2 Mb	PacBio Revio; AVITI	[192]
	GCA_051122635.1	Yanbian cattle	2.9	86.4 Mb	PacBio HiFi; Hi-C	[193]
*Bos indicus*	GCA_000247795.2	Nelore	2.7	28.4 Mb	SOLiD	[194]
	GCA_030271795.1	Wenshancattle	2.7	66.8 Mb	PacBio; Illumina HiSeq	[67]
	GCA_030269815.1	Leiqiong cattle	2.7	54.8 Mb	PacBio; Illumina HiSeq	[67]
	GCA_030271805.1	Weizhou cattle	2.7	38.7 Mb	PacBio; Illumina HiSeg	[67]
	GCA_030270715.1	Guanling cattle	2.7	12.8 Mb	PacBio; Illumina HiSeg	[67]
	GCA_029378745.1	SahiwalxTharparkar	2.7	42.1 Mb	PacBio	USDA ARS
	GCA_002933975.1	Gir	2.7	64.5 Mb	454; IonTorrent; IlluminaNextSeq; Illumina MiSeq	Anand Agricultural University
	GCA_963966425.1	Red sindhi	2.7	199.1 Kb	10× Genomics Chromium; Illumina HiSeq X	[195]
	GCA_963966355.1	Sahiwal	2.7	178.5 Kb	10× Genomics Chromium; Illumina HiSeq X	[195]
	GCA_963966215.1	Kankrej	2.7	164.7 Kb	10× Genomics Chromium; Illumina HiSeq X	[195]
	GCA_963966345.1	Tharparkar	2.8	126.3 Kb	10× Genomics Chromium; Illumina HiSeq X	[195]
	GCA_963966175.1	Gir	2.8	106.3 Kb	10× Genomics Chromium; Illumina HiSeq X	[195]
*Bos mutus*	GCA_027580195.2	Yak	2.6	38.3 Mb	Nanopore; Hi-C; Illumina	[196]
	GCA_002968435.1	Yak	2.3	23.6 Mb	Illumina HiSeq; Illumina GA	Institute of Bioinformatics and Applied Biotechnology
	GCA_007646595.3	Datong Yak	2.8	90.6 Kb	Illumina HiSeq	Lanzhou University
*Bos javanicus*	GCA_032452875.1	Banteng	3	47.1 MB	Oxford Nanopore	Oklahoma State University
*Bos gaurus*	GCA_014182915.2	Gaur	2.7	13.3 Mb	PacBio	USDA, ARS, USMARC
	GCA_965225615.1	Gaur	2.6	323.2 Kb	-	The University of Queensland
*Bos frontalis*	GCA_043643345.1	Chinese Gayal	2.6	20.2 Mb	PacBio RSII	[197]
	GCA_007844835.1	Gayal	3	28.7 Kb	Illumina HiSeq; PacBio	[198]
	GCA_017311355.1	Gayal	2.8	9.6 Kb	-	Bangladesh Livestock Research Institute; Chittagong Veterinary and Animal Sciences University

In conclusion, the bovine pangenome represents a paradigm shift from a static reference to a dynamic, population-scale representation of diversity. Future breeding programs must move beyond SNP-based selection to integrate graph-based SV genotyping. This will enable the precise utilization of “hidden” genetic variations. Meanwhile, the Cattle Genome-Tissue Expression Atlas (CattleGTEx) project can be fully utilized to elucidate the molecular regulatory mechanisms underlying important economic traits in domestic cattle [199], and techniques like CRISPR/Cas9 could introduce heat-tolerance alleles from zebu breeds into high-producing Holstein genomes, creating “climate-smart” cattle without the need for decades of backcrossing [200,201].

## 7. Search Strategy

To ensure a comprehensive synthesis of the genomic landscape of cattle, a systematic literature search was conducted, focusing primarily on articles published between 2010 and 2026. Peer-reviewed literature was retrieved from major scientific databases, including PubMed, Web of Science, and Google Scholar. The search strategy employed a combination of Boolean operators and the following keywords: “cattle”, “bovine”, “*Bos taurus*”, “*Bos indicus*”, “taurine”, “indicine”, “domestication”, “aurochs”, “dispersal”, “genomes”, “environmental adaptability”, “adaptive evolution”, “heat tolerance”, “cold adaptation”, “high-altitude adaptation”, “parasitic disease resistance”, “artificial selection”, “temperament”, “behavior”, “lactation traits”, “meat traits”, “pangenome”, and “structural variation”. During the literature screening process, inclusion criteria prioritized high-impact original research articles, comprehensive meta-analyses and reviews, and studies utilizing high-density SNP arrays and whole-genome sequencing. Studies were strictly selected based on their relevance to the evolutionary history, geographic dispersal trajectories, and genomic adaptation mechanisms of *Bos taurus* and *Bos indicus*. Additionally, classical archaeological findings and foundational paleogenomic studies predating 2010 were included to provide the essential historical and evolutionary context required for this review.

## 8. Conclusions

The evolutionary trajectory of domestic cattle stands as a testament to the power of human–animal mutualism. From the initial domestication of the aurochs to their widespread dispersal across every continent, cattle have continuously adapted to novel environments through a combination of standing genetic variation, adaptive introgression from wild relatives (such as banteng and yak), and intense artificial selection. By synthesizing robust evidence from archaeology and molecular biology, we have reconstructed the two independent domestication events of taurine and zebu cattle and traced their complex migration and hybridization history. Moving forward, research into the origins of domestic cattle should increasingly focus on historical convergence zones, particularly in Southeast Asia, to comprehensively map the distribution of local aurochs and the intricate pathways of early cattle introductions. Overall, the bovine genome has evolved a versatile genetic foundation, driven by both natural and artificial selection, enabling cattle to meet diverse production objectives across contrasting environments. As genomic research firmly transitions into the pangenome era, the high-resolution analysis of structural variations will unlock a more profound understanding of hidden genetic diversity. This paradigm shift will provide critical, previously inaccessible genomic resources for future precision breeding, ensuring the development of resilient breeds capable of thriving amidst global environmental changes.

## Figures and Tables

**Table 1:** Domestic cattle populations classified based on uniparental and autosomal markers.

Major Uniparental Haplogroup and Autosomal Ancestry		Primary Distribution	Evolutionary Significance
Mitochondrial haplogroups	T (T1–T3)	Ancient Near East, Global	The signature of taurine cattle domestication in the Near East [5]
	T1	Africa	Dominant in African cattle [38]
	T2, T3	Europe, Near East	T3 is the ubiquitous European lineage; T2 is common in the Near East [27]
	T4	East Asia	A derived sub-branch of T3, specific to East Asian taurine breeds [33]
	I (I1, I2)	South Asia	The signature of zebu cattle domestication in Indus Valley [6]
	P, Q, R, C	Ancient Europe and Asia	Rare lineages, representing ancient introgression from wild aurochs [14,39,40]
Y chromosome haplogroups	Y1	Northern Europe	Represents hybridization with European wild aurochs bulls [34]
	Y2 (Y2a, Y2b)	Eurasia, Africa	The primary paternal lineage of Near Eastern taurine cattle [9,34]
	Y3 (Y3a, Y3b)	South Asia, Africa	The primary paternal lineage of zebu cattle [9,36,37]
Autosomal ancestral groups	Taurine ancestry (European taurine, East Asian taurine, African taurine)	Eurasia, Africa	The taxonomy and distribution of modern taurine cattle [9,31]
	Indicine ancestry (South Asian indicine, East Asian indicine, African indicine)	South Asia, East Asia, Africa	The taxonomy and distribution of modern indicine cattle [10]

**Table 2:** Genes under selection in different cattle breeds.

Breed/Population	Trait	Associated Traits or Gene Functions	Candidate Genes	Statistical Test	References
Boran, Ogaden, Kenana	Coat color	Light coat color	*MLPH*, *SLC45A2*	XP-EHH, XP-CLR	[81]
Ankole	Coat color	Red, white-spotted	*MC1R*, *KIT*	XP-EHH, XP-CLR	[82]
Leiqiong cattle	Coat color	Black, yellow	*CORIN*	GWAS, F_ST_, π, Tajima’s D	[83]
Sumatran native cattle	Coat color	Brown, black, white	*CYFIP2*, *SGSM1*	GWAS	[84]
Zhoushan cattle	Coat color	Dark black	*MC1R*	F_ST_	[85]
Nguni	Coat color	Black, red	*MC1R*	GWAS	[86]
Nellore	Coat color	Darker hair on the head, neck, hump and knee regions	*ASIP*	GWAS	[87]
Holstein–Friesian mixed-breed	Coat color	White spotting	*KIT*, *MITF*, *PAX3*	GWAS	[88]
Tibetan cattle	Coat color	Gray	*KIT*	SNP-F_ST_, SNP-GWAS, SV-F_ST_, SV-GWAS	[89]
Hereford, Kazakh Whiteheaded	Cold climate adaptation	Cold-stress, thermoregulation, fat thermogenesis	*GRIA4*, *COX17*, *MAATS1*, *UPK1B*, *IFNGR1*, *DDX23*, *PPT1*, *THBS1*, *CCL5*, *ATF1*, *PLA1A*, *PRKAG1*, *NR1I2*	F_ST_	[90]
Hereford, Kazakh Whiteheaded	Cold climate adaptation	Cold shock response, thermoregulation	*MSANTD4*, *GRIA4*	GWAS	[91]
Russian cattle	Cold climate adaptation	Cold stress, nutrition balance	*AQP5*, *RETREG1*, *RPL7*, *TNKS*, *CERKL*, *HDAC3*, *ARRDC3*	DCMS	[92]
Yanbian cattle	Cold climate adaptation	Cold stress, hair development, lipid metabolism	*CORT*, *FGF5*, *CD36*	CLR, θπ, XP-CLR, F_ST_, θπratio	[93]
Mongolian cattle, Yanbian cattle	Cold climate adaptation	Lipid metabolism, oxidative phosphorylation	*UQCR11*, *DNAJC18*, *EGR1*, *STING1*	FLK, hapFLK	[94]
Apeijiaza cattle, Anxi cattle	High-altitude environments adaptation	Hypoxia adaptation, cardiovascular metabolism	*NOXA1*, *RUVBL1*, *SLC4A3*, *LETM1*, *TXNRD2*, *STUB1*	CNV-F_ST_	[95]
Zhangmu cattle, Anxi cattle, Qaidam cattle	High-altitude environments adaptation	Hypoxia adaptation	*PPARGC1A*, *ANGPT1*	F_ST,_ Tajima’s D, π-ratio, V_ST_	[96]
Zhangmu cattle, Anxi cattle, Qaidam cattle	High-altitude environments adaptation	Hypoxia adaptation, immunity, lipid metabolism	*PTPN9*, *IL6*, *B4GALNT1*, *PLIN2*, *NOS2*, *EPAS1*, *EGLN1*	θπ, F_ST_, Tajima’s D, rIBD	[97]
Tibetan cattle	High-altitude environments adaptation	Energy metabolism erythropoiesis, angiogenesis, peroxisomal metabolism	*EGLN1*, *PPP1R14C*, *NFE2L2*	SV-F_ST_	[89]
Tibetan cattle	High-altitude environments adaptation	Hypoxia response, cold adaptation	*EGLN1*, *LRP11*, *LATS1*, *GNPAT*	F_ST_, θπ, Tajimas’ D, TreeMix, D statistic	[98]
Gojjam	High-altitude environments adaptation	HIF1 signaling pathway, antioxidant	*GBE1*, *NEK7*, *SOD1*	CNV-V_ST_	[99]
Boran, Kenana, Ogaden	Thermotolerance	Oxidative stress response	*HSPA4*, *SOD1*, *PRLH*	XP-EHH, XP-CLR	[82]
Tharparkar, Gir, Ongole	Thermotolerance	Heat stress	*HSPA1B*, *HSPA12A*, *GRXCR1*, *FKBP4*, *HSPA1L*, *IL6*	iHS, ROH, F_ST_	[100]
East Asian indicine cattle, South Asian cattle	Thermotolerance	Heat tolerance, immunity, light-coated coat	*DNAJC18*, *HSPA9*, *MATR3*, *MZB1*, *STING1*, *LIPH*, *FGF22*, *LEF1*, *ASIP*, *TRPA1*, *ILDR*, *CASR*, *HBA*, *HBA1*, *HBQ1*, *HBM*	F_ST_, π ratio, XP-EHH, CLR, iHS, D and f3 statistics	[10]
Hainan cattle	Thermotolerance	Heat tolerance	*CRNN*, *SBSN*, *SPINK5*	DI_SV_, SV-F_ST_	[101]
Hainan cattle	Thermotolerance	Heat tolerance, inflammation	*DNAJC3*, *HSPA1A*, *CD53*, *ZBTB12*, *AHCYL2*	CLR, Tajima’s D, ROHs	[102]
Abigar, Fellata	Thermotolerance, tick resistance	Heat stress, heat shock protein, immune response	*HSPA13*, *DNAJC18*, *DNAJC8*, *KRT33A*, *BoLA*	CNV-V_ST_	[99]
African cattle	Tick resistance	Antigen recognition	*BoLA*	XP-EHH, XP-CLR	[82]
Nguni	Tick resistance	Keratin, heat resistance	*KRT222*, *KRT24*, *KRT25*, *KRT26*, *KRT27*, *HSPB9*, *CYM*, *CDC6*, *CDK10*, *KCNBI*, *TNS4*	F_ST_	[103]
Boran, Ogaden, Kenana	Tick resistance, parasite resistance	Antigen recognition and processing, keratin and epidermis, gastrointestinal immunity	*BoLA*, *SLC25A48*, *KRT33A*, *MC5R*, *TGM1*, *TGM3*, *DMBT1*	XP-EHH, XP-CLR	[81]
N’Dama	Trypanosomosis resistance	Feeding and drinking behaviors, Anemia, iron homeostasis	*HCRTR1*, *SLC40A1*, *STOM*, *SBDS*, *EPB42*, *RPS26*	XP-EHH, XP-CLR	[82]
Sheko	Trypanosomosis resistance	Anemia, immune tolerance, neurological dysfunction	*MIGA1*, *CDAN1*, *HSPA9*, *PCSK6*, *SPAG11B*, *RAETIG*, *PPP1R14C*, *TTC3*, *ERN1*, *CAPG*, *TTBK2*, *POLR3B*, *GNAS*	iHS, Rsb, CLR	[104]
Leiqiong cattle, Lufeng cattle	Parasite resistance	Immunity	*BCAR3*, *PRNP*, *TRAPPC9*, *TMSB4*, *TGM3*, *LTF*	iHS, Rsb, F_ST_	[105]

## Data Availability

No new data were created or analyzed in this study.
